# Materia Medica Study

**Published:** 1891-10

**Authors:** W. A. Yingling

**Affiliations:** Nonchalanta, Kansas


### MATERIA MEDICA STUDY.

W. A. Yingling, M. D., Ph. D., Nonchalanta, Kansas.

Whilst man is the highest type of the animal creation, yet he is a creature of circumstances requiring development to bring forth that which distinguishes him from the inferior animals. Man alone must be qualified to occupy the position for which he was made. By nature he is adaptable, but never adapted for his sphere in life without training and considerate guidance. Abraham Lincoln stands as the model of adapted humanity; the street urchin as the adaptable. The new-born babe is but a germ in all its faculties and intellectual powers. Vast possibilities are before it, but the goal of true manhood is never reached without the requisite developmental adaptability. It must be made a man. It will grow physically, and the senses will be more or less developed, but to reach the height of intellectual manhood the germ, the embryonic mind, must be trained and carefully nurtured.

The five senses of human nature are the media of this adaptability; they are but embryonic and require development, but through them the germ-mind is brought into full existence and led to the power of abstraction and scientific and philosophic knowledge. Without these five senses the child would be absolutely isolated from the rest of the universe; without any one of these senses there would be absolute ignorance of all knowledge derived by that sense. The congenitally blind can have no true conception of color because they have no knowledge of color derived by the faculty of perception through the sense of sight. Their understanding gives no response to the words used to express the idea of color. They may use the words, and even speak of the blending of colors intelligently, but their mind forms no mental picture corresponding to the true conception of color as with those who have perceived colors by the sense of sight.

The mind is always led from the known to the unknown; from the tangible to the intangible; from the concrete to the abstract. To conceive properly we must first perceive. Perception is the result of one of the disturbed senses on the mind; conception is an act of the mind itself.

In further aid of this development of the man as he should be, we have a faculty whereby, or by the power of which, the impressions and ideas brought into the mind by perception and conception are stored away for future use and brought up again whenever occasion demands. This faculty is called memory, and is that faculty least understood without proper consideration, and the one most essential to the successful homœopathician. It is the one faculty that makes the ready use of the vast materia medica possible to the busy physician at the bedside. Judgment and discernment are *sine qua nons*, essentials, but the ready memory is that faculty so much needed at times when we cannot sit down in our *otium cum dignitate* and leisurely discern and judge of the required remedy. The three essentials of an expert physician, outside of moral character and common sense, are judgment, discernment, and a reliable and ready memory.

Memory gives us a notion of time and duration; without it there would be no yesterday and no thought of the past. It is also the basis of experience, and consequently of all progress. Take from the physician memory and he could not build upon his past failures and success. Each case would be the same—a new one for present consideration. By the aid of memory the physician builds upon experience and advances the medical science to the ideal of comparative perfection. Hence, it is a duty of the doctor to improve his memory, and thus give his patients the benefit of the experience of himself and others.

We have two kinds of memory, the Spontaneous and the Intentional. The Intentional memory is a *re*-collection of the impressions of the mind and may be difficult because the concepts have not been definite nor vivid. The Spontaneous memory is the kind to be acquired by the physician. The spontaneity of the act of remembering will be proportionate to the vividness of the mental picture formed by the concept, hence a way must be sought out by which a mental picture may be formed so vividly as to recall the information needed promptly and certainly.

To trust the memory is to strengthen it, because this trust exercises it, and thus develops it. The blacksmith's arm becomes strong because he uses it, and he uses it because he trusts it. If he had no faith in his muscles he would not exercise them. By his faith he puts forth the effort, and in time, according to law, he possesses a strong and skillful arm.

Mnemonics, or Memoria Technica, is the artificial method and rests exclusively on the association of ideas. This aid to the memory has been traced back to Simonides, in the sixth century B. C. Cicero, Quintilian, and Pliny, the Naturalist, and many others of the ancients, also made good use of some form of Mnemonics. Among the moderns, who practiced and taught this art, may be mentioned Gray, Feinagle, Loisette, and many others. The last, perhaps, being the best, but all are too cumbersome for the busy practitioner. There is a more direct way—that is, to adopt the plan of mental picture-making in accordance with the well-known law of association, which will produce spontaneity in recalling the facts to mind as they are needed.

Locke says, "Ideas that in themselves are not all of kin, come to be so united in some men's minds that it is very hard to separate them; they always keep company, and the one no sooner at any time comes into the understanding but its associate appears with it."

Kant says, "The law of *association* is this—That empirical ideas which often follow each other, create a habit in the mind, whenever the one is produced, for the other always to follow."

I need not go into further detail to show what I wish to bring before the mind of the readers of THE HOMŒOPATHIC PHYSICIAN. It is simply to make use of the law of association in the study of our mammoth materia medica. To impress the utility of this plan on the reader's mind we hint at several facts, and then give our plan. Our space is too circumscribed to go into detail.

Whenever a disease is cured by a given remedy the remedy is fixed in the mind and thereafter the same disease, like circumstances, will recall the same remedy. The picture of the diseased condition and the remedy are associated together; the picture must present the remedy to be complete. This is called experience. It is the recognition of this fact that causes so many to prefer the physician of experience to the young man without experience. In the old school this is a requisite, but in Homœopathy, whilst a decided advantage, yet having a true and fixed law of cure, the young man with a discerning and comprehending understanding may be the better prescriber.

A remedy curing a disease fixes its remedial action in the mind. When the remedy comes before the mind the diseased condition also presents itself. This aids in the abstract study of the materia medica; the former statement aids in the therapeutical study of the remedies. Both are essential, and in accordance with the law of the mind known as association.

I mention the name of U. S. Grant. Those who have seen him at once have a mental picture of him as they saw him, or as they saw him under the most impressive circumstances; those who have never seen him at once recall some circumstance of him that was most impressive to them. No doubt Lee had a mental picture of Appomatox whenever he heard the name of Grant, because the most impressive to the old veteran. Thus vividness and impressiveness are two of the characteristics of this law of association. Thus, by this law, the impressions of childhood, the old home and fields and woods, the faces of dear ones and companions, are brought suddenly to mind when some one mentions a circumstance or a familiar name associated with early childhood. How readily the name calls to mind the form, characteristics, or peculiarities of the person bearing the name, and every time the most peculiar characteristics come first, because most vividly impressed. Mr. A. had a cancer that entirely destroyed his eyes and nose; you had often seen him and was impressed deeply; mention Mr. A.'s name and how readily that picture presents itself. Or mention the name of the disease, and the appearance, etc., of Mr. A. are introduced. We are all more or less governed by mental pictures imprinted upon the mind unwittingly.

Our plan in the study of the materia medica is to form a vivid mental picture of the symptoms of the given remedy. To so closely connect the remedy with certain conditions that when you see the condition the remedy inevitably presents itself to the mind—the more vivid the picture the more distinctly the remedy comes to mind. Then have the picture so accurately drawn by close study and *distinction between remedies* that you may be positive that the association will reproduce the remedy. Trust your memory by carefully giving it the qualifications of credibility. This may be done by the care taken in the artistic mental picture. If one is unable to form a vivid picture by the disturbance of other thoughts, or by the diversion of his mind by mental wandering, he must blame his weakness and not censure his ever trusty memory, or else seek the simillimum to restore his mind to a healthy state. When the mind is sound, the picture accurate and vivid, the result will be in accordance with this mental law. Read the two quotations above, from Locke and Kant.

To be more particular: You are studying the action of *Lach.* in ulcers. Form a mental picture of an ulcer, just the one calling for Lachesis, and associate the remedy with that particular kind—*see* the hard circumference, the patient cringing from sensitiveness of the sore, the black bottom, easily bleeding, but very little pus. This is to be done with all the remedies in our study. It is easier than to endeavor to recollect, because the association gives spontaneity to the action of the memory. It requires but little time, and with the habit formed the process will become almost instantaneous. Then, this habit must be carried to the sick room. You see the patient suffering from terrible pain; a certain remedy magically relieves. Carry that exact picture and the remedy in the same recess of the mind.

Some may object that too many pictures must be formed, and that confusion would be the result. Confusion will be the result of incomplete mental concepts only. Complete pictures increase the mental power as each stroke of the hammer strengthens the arm of the smith. Use strengthens the memory; there is no gorging of this faculty when the law of association is observed, and when vividness and completeness are the artists. The memory is capable of wonders, seemingly miraculous feats, and there seems to be no limit to its healthy exercise. Call to mind all the articles, persons, subjects, etc., an ordinary person knows and can name at sight. To what greater extent does the mind of the professional man go? There really seems to be no limit to the powers of the memory.

Others may object that this plan requires too much time. Time only is required in forming the habit, then it is almost instantaneous. Note the feat of memory of various persons who can name distinctly and readily a large number of articles promiscuously arranged in a show-window by a mere glance as they rapidly walk by. This is mere habit of the memory secured by a practiced perception—a trained eye and an instantaneous mental picture.

By proper training and direction the sensitive brain of the physican can record the pictures of the remedies so as to be spontaneously reproduced when needed. Poor memories are usually the result of the want of attention which prevents vividness. A good memory is the result of a vivid and clear picture rapidly, or instantaneously, imprinted on the brain. It is to be secured by practice and consequent habit. As soon as the habit is formed, experience in picture forming will give rapidity in both the recording and the reproducing of the data desired. Why is it that the man who has the so-called poor memory for faces can, with a single glance, vividly recall the face of the villain who assaults him, and that face is before him at every alarm? Here fear, riveted attention, and hyperæsthesia of the mind produced by alarm, indelibly imprint the likeness on the brain. Interest in the theme, a sense of duty to the sick, and the habit of close attention should produce the same vivid picture on the brain of the homœopathician.

